# The Geometry of Nanoparticle-on-Mirror Plasmonic Nanocavities Impacts Surface-Enhanced Raman Scattering Backgrounds

**DOI:** 10.3390/nano14010053

**Published:** 2023-12-24

**Authors:** Zixin Wang, Wenjin Zhou, Min Yang, Yong Yang, Jianyong Hu, Chengbing Qin, Guofeng Zhang, Shaoding Liu, Ruiyun Chen, Liantuan Xiao

**Affiliations:** 1 State Key Laboratory of Quantum Optics and Quantum Optics Devices, Institute of Laser Spectroscopy, Shanxi University, Taiyuan 030006, China; 2 Collaborative Innovation Center of Extreme Optics, Shanxi University, Taiyuan 030006, China; 3 Key Laboratory of Advanced Transducers and Intelligence Control System, Ministry of Education, Taiyuan University of Technology, Taiyuan 030024, China; 4 College of Physics, Taiyuan University of Technology, Taiyuan 030024, China

**Keywords:** surface-enhanced Raman scattering, background, self-assembled monolayer, nanoparticle on mirror, plasmonic nanocavity, dark-field scattering

## Abstract

Surface-enhanced Raman scattering (SERS) has garnered substantial attention due to its ability to achieve single-molecule sensitivity by utilizing metallic nanostructures to amplify the exceedingly weak Raman scattering process. However, the introduction of metal nanostructures can induce a background continuum which can reduce the ultimate sensitivity of SERS in ways that are not yet well understood. Here, we investigate the impact of laser irradiation on both Raman scattering and backgrounds from self-assembled monolayers within nanoparticle-on-mirror plasmonic nanocavities with variable geometry. We find that laser irradiation can reduce the height of the monolayer by inducing an irreversible change in molecular conformation. The resulting increased plasmon confinement in the nanocavities not only enhances the SERS signal, but also provides momentum conservation in the inelastic light scattering of electrons, contributing to the enhancement of the background continuum. The plasmon confinement can be modified by changing the size and the geometry of nanoparticles, resulting in a nanoparticle geometry-dependent background continuum in SERS. Our work provides new routes for further modifying the geometry of plasmonic nanostructures to improve SERS sensitivity.

## 1. Introduction

Metallic nanostructures possess the ability to confine light below the diffraction limit via the excitation of localized surface plasmons, which provides effective strategies for increasing the light–matter interaction. Extreme confinement of light allows for a dramatically enhanced electromagnetic field in the gap region between metallic nanostructures, making coupled metallic nanostructures a promising platform for nonlinear nano-optics [1,2], sensitive biosensing [3], and photocatalysis [4]. Among these nanostructures, the nanoparticle-on-mirror (NPoM) geometry [5], where a metal nanoparticle is separated from a metal film via a self-assembled molecular monolayer or a thin layer of 2D material, has attracted great attention due to its ease of fabrication but strong light–matter coupling. Strong light–matter interaction due to plasmon coupling can enhance the weak optical processes of materials in the nanocavity, which has been utilized to amplify second-harmonic generation [6], single photon emission [7], upconversion luminescence of lanthanide-doped nanoparticles [8,9], and surface-enhanced Raman scattering (SERS) from molecules [10]. Recently, the SERS of molecules in plasmonic nanocavities has received much attention because of its ability to upconvert mid-infrared radiation to visible light [11,12,13]. However, despite huge enhancements in Raman scattering, the interaction of metal nanostructures with molecules may also influence the molecular vibrations and therefore the optical behaviors. One of the influences is the appearance of a background in the SERS, which will ultimately impair its sensitivity.

Many efforts have previously been devoted to studying the origin of this background [14,15,16,17,18]. The first assumption is the contribution of SERS from contaminant molecules on the metal nanostructures. However, some reports ruled this out by changing the substitute molecules on metal surface [18] or by using pure metal [14]. Another possibility is the light emission from the metal [17,19]. While the photoluminescence of metal nanostructures can be detected when excited with light at a wavelength much shorter than their plasmon resonance, there is no considerable evidence for light emission of metal excited at a much longer wavelength where the scattering of molecules can still be detected. Instead of emissions from interband transition from the *s*-band to *d*-band in metal [20,21], Hugall et al. proposed that the light emission of Au when excited with a longer wavelength comes from inelastic light scattering from the electrons in metal nanostructures, which contributes to the background continuum in SERS [16]. However, information regarding interactions was derived from experiments conducted on a large ensemble of molecules and metal films. Although ensemble-averaged results are essential, they often preclude local parameters of the dynamic process. Typically, the optical properties of plasmonic nanocavities can be influenced by the size [22,23] and geometry [24,25] of the nanoparticle, as well as the variation of gaps [26,27] in the nanocavity. The background continuum in SERS may depend on the geometry of the nanocavities in ways that are not yet well understood. Another factor that may be important is that the variation in the self-assembled monolayer under laser irradiation will change the stability of NPoM cavities [28,29], which thus may modify the background of SERS measurement.

Here, we systematically study the effect of nanocavity geometry on the SERS of biphenyl-4-thiol (BPhT) molecules within NPoM plasmonic nanocavities. We fabricate NPoM nanocavities with distinct nanoparticle geometry, including Au nanosphere-on-mirror (NSoM) and Au nanocube-on-mirror (NCoM), with different cube sizes. We investigate the nanoparticle geometry-dependent changes of the SERS signal and background induced by laser irradiation on the molecular monolayer with distinct conformation, in which the molecule conformation is controlled by changing the incubation time (2 h and 24 h) of the mirror in BPhT solution. To remove ensemble averaging, the Raman scattering from molecules within individual NPoM plasmonic nanocavities is monitored separately, while simultaneously characterizing the properties of NPoM plasmonic nanocavities by using dark-field scattering spectroscopy and scanning electron microscopy (SEM). Our study reveals the critical role of the geometry of nanostructures in influencing the SERS signals of the molecular monolayer and the background continuum in NPoM plasmonic resonators. It should stimulate further efforts in controlling the geometry of plasmonic nanostructures in order to inhibit continuous backgrounds which are detrimental to the sensitivity of sensing based on SERS.

## 2. Materials and Methods

The Au nanospheres and nanocubes capped with cetyltrimethylammonium bromide (CTAB) were purchased from a commercial supplier (Nanoseedz Inc., Hong Kong, China). The nanospheres had a size distribution centered at 119.49 nm with a width of 10.84 nm. The average edge length of the Au nanocubes for each sample was determined by evaluating the SEM images (Figure S1), which showed a size distribution centered at 60.81 nm, 81.90 nm, and 92.43 nm, respectively (Figure S2).

The NPoM samples were prepared on a silicon substrate (500 μm thick) with micro-markers prefabricated to provide a reference for the position of the nanoparticles. First, the precleaned substrates were covered with a 5 nm Cr layer and a 50 nm Au layer by using an evaporator. The substrates covered with the Au film were then separated into two groups which were immersed in a 1 mM solution of BPhT (Sigma-Aldrich, St. Louis, MO, USA, 97%) dissolved in ethanol for 2 h and 24 h, respectively. After the immersion, the samples were rinsed with ethanol and dried. Finally, a thin film of self-assembled molecular monolayer was deposited on the Au film. Plasmonic nanocavities were fabricated by drop-casting a small amount of each colloidal Au nanoparticle solution onto the Au film, resulting in Au nanoparticles randomly distributed on the monolayer-covered Au films. 

SERS and dark-field scattering were carried out using home-built setups based on an optical microscope (ECLIPSE TE2000-U, Nikon, Minato City, Tokyo, Japan) and a dark-field microscope (BX53, Olympus, Shinjuku City, Tokyo, Japan), respectively. To detect the SERS (Figure S3), the NPoM sample was placed on a nanopositioner (Tritor 200/20 SG, Piezosystem jena, Jena, Germany). A 785 nm diode laser (MDL-E-785 nm, Changchun New Industries Optoelectronics Tech. Co., Ltd., Changchun, China) was used for excitation. For SERS imaging and spectroscopy, the laser was focused by an objective lens (Plan 100×/0.9 NCG, Nikon, Minato City, Tokyo, Japan) onto the sample. The scattered light collected by the same objective passed through a dichroic mirror (NFD01-785-25×36, Semrock, Rochester, NY, USA) and was filtered by a notch filter (BSP01-785R-25, Semrock, Rochester, NY, USA), which was then detected by a single photodetector (SPCM-AQR-15, PerkinElmer, Waltham, MA, USA) and a spectrograph (SR-500i-A, Andor, Oxford Instruments, Abingdon, UK) equipped with an EMCCD (Newton, DR-316B-LDC-DD, Andor, Oxford Instruments, Abingdon, UK). To achieve the Stokes sideband of the SERS, the scattered light was filtered by a long-pass filter (Di02-R785-25×36, Semrock, Rochester, NY, USA). For dark-field imaging and spectroscopy (Figure S4), a halogen lamp (U-LH100L-3, Olympus, Shinjuku City, Tokyo, Japan) was used for illumination. After passing through an aperture with a light stop, the white light from the halogen lamp was focused by an objective lens (MPlanFL N 100×/0.90 BD, Olympus, Shinjuku City, Tokyo, Japan) for dark-field illumination. The reflected light from the sample was then detected with a color camera (U-TV1XC, Olympus, Shinjuku City, Tokyo, Japan) for imaging and with a spectrometer (PI-HRS300, Princeton Instruments, Trenton, NJ, USA) equipped with a CCD (ProEM HS:1024BX3, Princeton Instruments, Trenton, NJ, USA) for dark-field spectroscopy.

## 3. Results and Discussion

**Figure 1:**
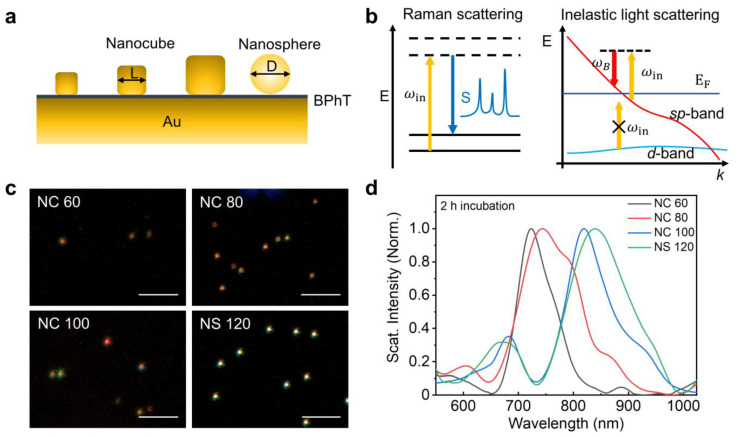
(**a**) Schematic view of NPoM cavities formed by Au nanocubes with different-side-length L and Au nanospheres with diameter D over an Au film of 50 nm. The nanoparticles are separated by a thin layer of self-assembled BPhT molecules from the Au film; (**b**) Schematic of the Raman process in molecules and the origin of the background; (**c**) Dark-field scattering images of NPoM nanocavities formed with Au nanocubes with average side lengths of approximately 60 nm, 80 nm, and 100 nm, and Au nanospheres with a diameter of around 120 nm. Scale bar is 5 μm; (**d**) Typical dark-field scattering spectra of NPoM nanocavities with gap spacer of self-assembled BPhT monolayer prepared with 2 h incubation time.

The geometry of the final NPoM system is shown in Figure 1a. Nanocubes with three different side lengths (termed NC 60, NC 80, and NC 100) and nanospheres (termed NS 120) were deposited onto a Au film of 50 nm to form the nanocavities. BPhT monolayers formed upon two different incubation times, i.e., 2 h and 24 h, provide nanometer-thick spacers with different conformations [28]. To characterize the influence of the nanoparticle geometry on the optical properties of NPoM systems, both the SERS enhancement of the BPhT monolayer and the dark-field scattering properties were studied. From the dark-field imaging of nanocavities (Figure 1c), we can find that the nanoparticles are dispersed individually on the gap spacer adsorbed on the Au film, allowing individual nanocavities to be probed. The dark-field image of NS 120 shows a higher intensity than any other three geometries, which can be attributed to the increase in the scattering cross-section with nanoparticle size [30,31,32]. Figure 1d shows typical dark-field scattering spectra of individual NPoM systems with variable nanoparticle geometry. At a fixed spacer thickness, NPoM nanocavities give scattering resonances which depend on the geometry and size of the nanoparticle. The dark-field scattering spectra of the NPoM plasmonic nanocavities show a strong peak at a longer wavelength and a weak peak at a shorter wavelength, which are commonly ascribed to the bonding dipole mode from plasmon coupling of the nanoparticle and the mirror and the transverse cavity mode, respectively [24,33]. The transverse cavity mode is also pronounced in the NS 120 sample due to the existence of a facet on the nanosphere [23]. Moreover, we notice that some higher-order modes also appear due to the plasmon hybridization in the nanocavities [24,33]. Red shifts of the plasmon resonance are seen with the increasing size of the nanocube. This can be attributed to the increase in the length of the cavity, which will support a resonance at a longer wavelength [27]. 

**Figure 2:**
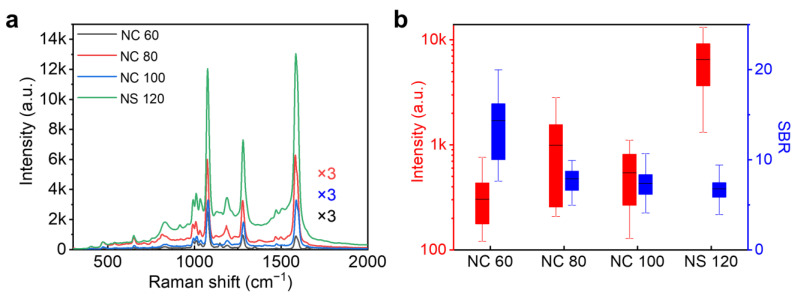
(**a**) SERS spectra of BPhT monolayer in the NPoM geometry with different nanoparticle sizes and geometries. (**b**) SERS intensity and signal-to-background ratio extracted at 1078 cm^−1^ versus nanoparticle size.

Figure 2 shows typical SERS spectra of BPhT molecules in NPoM nanocavities. The laser power was set to 1.38 mW. The SERS spectra of BPhT molecules are highly reproducible among different nanocavities, which show strong vibrational peaks at 1078 cm^−1^, 1281 cm^−1^, and 1586 cm^−1^, corresponding to the C-H bending and ring stretching in BPhT molecules [11,34]. To emphasize the changes caused by nanoparticle geometry, the spectra are averaged over many individual nanocavities for each size. Generally, the intensity of SERS shows increases with the increasing size of the nanoparticles [22]. However, we find that the increase in SERS signal for NCoM cavities is not linear. It appears that the SERS signal among NC 80 nanocubes is slightly higher than that of the other two sizes (Figure 2a), which can be attributed to the overlapping of the Raman laser with the plasmon resonance band of NCoM cavities. The SERS signal among NSoM cavities with 120 nm nanospheres shows a much higher peak intensity than that from the NCoM cavities, which indicates that there is a larger near-field enhancement in NSoM nanocavities.

Besides the peaks, continuous backgrounds were observed in each spectrum. To characterize the change in the continuous backgrounds with the geometry of the nanocavity, we focused on one of the strongest SERS peaks at 1078 cm^−1^. The peak intensity was extracted from the spectra directly while the background was achieved from the Gaussian fit of the continuum for each spectrum. Figure 2b shows the extracted peak intensity and the signal-to-background ratio (SBR) at 1078 cm^−1^ with the change of particle geometry and size. Surprisingly, even though the background continuum shows similar phenomena to the change of peak intensity as a function of nanoparticle size, we observe a clear anti-correlation between the peak intensity and the SBR, indicating that the background increases more than the SERS signal. Generally, as the number of molecules contributing to the SERS signal will increase with the increase in the cube length, one would expect enhanced SERS peaks accompanied by enhanced backgrounds. Thus, the deviation between the trend of background and SERS peak observed here indicates that the background does not originate from the molecular monolayer. Such an assertion can be further validated by looking at the background of SERS among 120 nm nanospheres over Au film. As a point-contact-type nanocavity, the NSoM geometry possesses a much smaller mode volume than the plan-contact-type, such as the NCoM structure [35]. This results in a lower number of molecules contributing to the SERS. However, the SERS signal among NS 120 shows a much higher peak intensity than that from the NCoM cavities, while its background does not increase proportionally.

Another significant contribution to the background may arise from light emission from the Au. Since the 785 nm Raman laser is well below the interband absorption from *d*-bands to *sp*-bands in Au [21], the continuous background cannot be the photoluminescence of Au nanoparticles. Instead, it has been suggested that the interaction between the incoming light and electrons in the plasmonic metals supports an inelastic light scattering [16] (Figure 1b) in which the electrons below the Fermi energy ($E_{F}$) are excited to a virtual state by the Raman laser ($\omega_{in}$) and then relax to *sp*-bands by emitting a red-shifted light ($\omega_{B}$), which closely resembles the Raman transition in molecules. However, compared to the Raman scattering process of molecules, relaxation of electrons to a different energy on the *sp*-band in metal nanoparticles always requires a change in their momentum [36]. Previous reports have suggested that the spatial localization of the plasmons can provide sufficient changes in the momentum of the electrons in metal [19,36], which can achieve an energy difference of up to 400 meV for 1 nm confinement of plasmon [16], allowing the possibility of a broadband background from inelastic light scattering.

**Figure 3:**
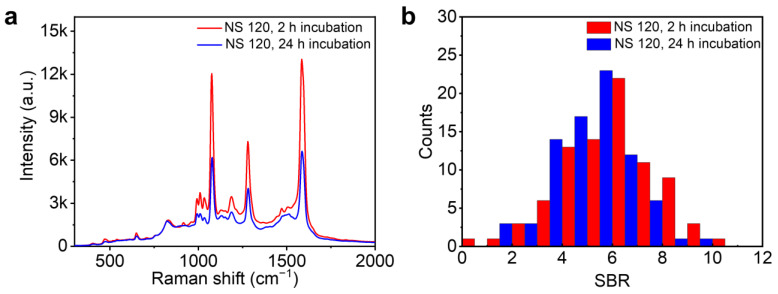
(**a**) SERS spectra for samples incubated for 2 h and 24 h, respectively, among NSoM cavities formed with 120 nm nanospheres. The spectra are averaged over SERS of BPhT molecules from at least 80 NSoM cavities. The laser power is set to 1.38 mW. (**b**) Distribution of the signal-to-background ratio for the SERS from BPhT with 2 h and 24 h incubation times.

Under these circumstances, plasmon coupling between the nanoparticles and the mirror will naturally influence the background continuum in SERS. The plasmon coupling in NPoM nanocavities depends on a number of factors, such as the dimensions and the geometry of the nanoparticle as well as the thickness of the spacer between the nanoparticle and the mirror. To investigate the role of plasmon in SERS background, we firstly compared the SERS from BPhT monolayers prepared with different incubation times (Figure 3). It has been reported that different incubation times can result in different monolayer morphologies [28]. The sample prepared with a longer incubation time of 24 h possesses densely packed and ordered molecules, leading to a larger gap size within NPoM plasmonic nanocavities, while with a shorter incubation time of 2 h, the molecules are sparsely packed and show a lying-down configuration, resulting in a smaller gap size. It can be found that the Raman scattering intensity from the sample with a shorter incubation time of 2 h is significantly higher than that from 24 h (Figure 3a). This can be attributed to the lager field enhancement in the gap region of NPoM nanocavities due to the decrease in gap distance [28]. Statistics on the SBR from all plasmonic nanocavities formed with 120 nm nanospheres show no significant change (Figure 3b), which means that the SERS background increases proportionally with the decrease in the gap distance. Nevertheless, the change in plasmon coupling in NPoM nanocavities solely by changing the thickness of the spacer cannot explain the dramatic increase in SERS background in NSoM nanocavities with 120 nm nanoparticles compared to the NCoM nanocavities shown in Figure 2. Furthermore, the distribution of SBR for the same sample also suggests that the geometry of the nanoparticle may play a more important role in determining the SERS background in the nanocavities. 

**Figure 4:**
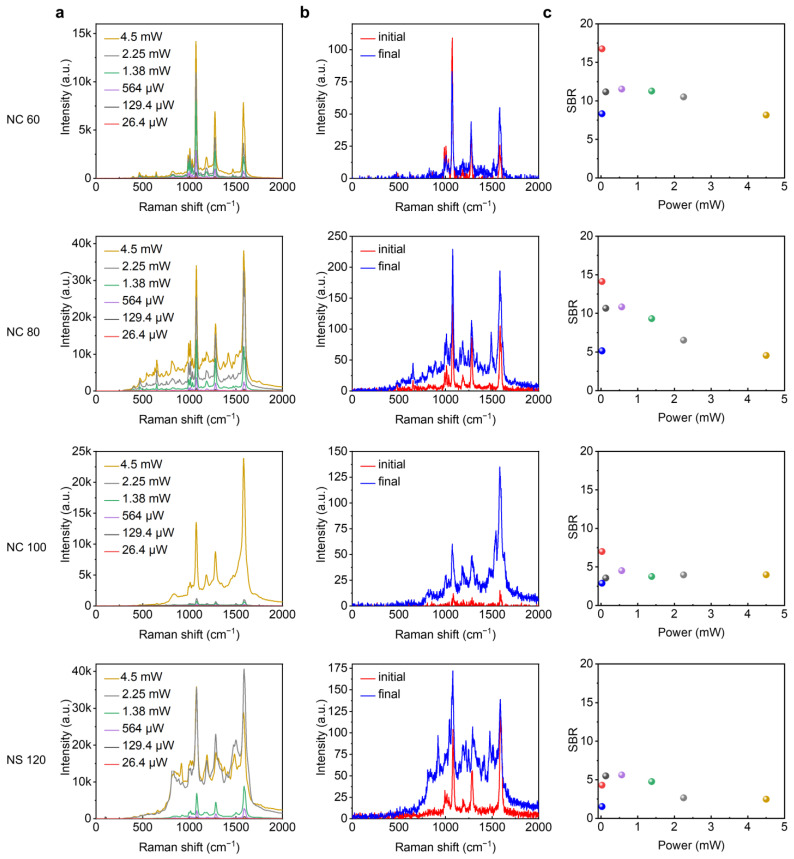
(**a**) Evolution of SERS spectra of spacer molecules among different NPoM systems upon laser exposure with increasing power for the sample prepared with incubation time of 24 h. (**b**) The SERS spectra for each sample achieved before and after laser exposure, with the same laser power of 26.4 μW. (**c**) Evolution of the corresponding SBR with laser power, where the blue circle indicates the SBR measured again using 26.4 μW laser power after one cycle of laser irradiation while the colors of the other circles correspond to that shown in (**a**).

To further verify the role of the geometry of the nanoparticles in SERS, we studied the evolution of SERS with increasing laser power. As the laser power was gradually increased, both the SERS intensity and background for samples among NPoM nanocavities with various geometries showed a progressive increase. However, the background among NPoM nanocavities with larger nanoparticle size showed a more significant increase with the laser power (Figure 4a and Figure S5). We find that the increase in background under laser irradiation is irreversible. Figure 4b shows the SERS spectra obtained before and after the laser irradiation process, under the same laser power of 26.4 μW. It can be found that the background continuum is maintained at a higher level than the initial level. The change is also more pronounced under larger nanoparticle size. Figure 4c shows the evolution of the corresponding SBR as a function of laser power. As the laser power further increases, the Raman scattering signal no longer increases. Instead, the background shows a further increase which lowers the SBR of the spectra (Figure 4c). Interestingly, it appears that there is an optimal power for each type of NPoM plasmonic nanocavity to achieve a higher SBR (Figure S6).

**Figure 5:**
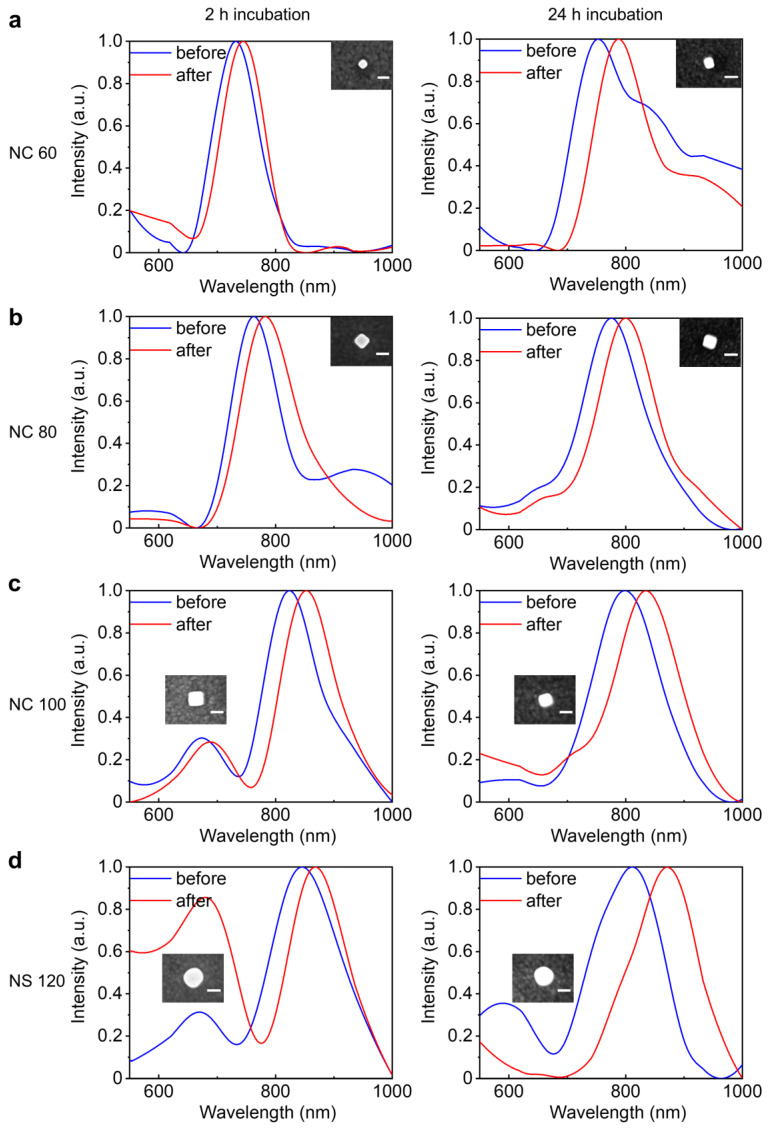
Evolution of the dark-field spectra of NPoM plasmonic nanocavities formed with nanocubes with sizes of approximately 60 nm (**a**), 80 nm (**b**), 100 nm (**c**), and 120 nm nanospheres (**d**) over monolayer molecules incubated for 2 h (**left column**) and 24 h (**right column**), respectively, upon laser exposure for 60 s with a power of 1.38 mW.

The laser-power-dependent change in SERS spectra observed here further highlights the influence of localized plasmons on the SERS signal and the background, partly due to the decrease in the gap distance within nanocavities. Recently, laser irradiation has been reported to induce changes in the conformation of molecules [28]. The height of the self-assembled monolayer decreases as the laser power increases, resulting in a smaller gap size and enhanced plasmon coupling between the nanoparticle and the mirror. Tighter plasmon localization can thus enhance the Raman scattering of spacer molecules in the NPoM nanocavities. The laser-induced change of plasmon coupling can be supported by the dark-field scattering spectra of the nanocavities. Figure 5 shows the dark-field scattering spectra of specific NPoM nanocavities measured before and after laser exposure. Although the profile of the dark-field spectra of NPoM nanocavities varies due to the distribution of the particle size, the dipole modes of all the nanocavities show a clear red shift upon laser exposure, the red shift is more pronounced for the sample with 24 h incubation time. We attribute this more pronounced red shift to the more significant decrease in gap distance in the nanocavities upon laser exposure. It has been reported that the sample prepared with a long incubation time of 24 h possesses a densely packed monolayer with molecules in an upright conformation [37]. Upon laser irradiation, the conformation of molecules will be changed from an upright configuration to a lying-down configuration [28]. This results in a decrease in the height of the spacer monolayer in the nanocavity, more prominent than the decrease in sample prepared with 2 h incubation which is already in a lying-down configuration. The decrease in the gap height will modify the plasmon coupling between the nanoparticle and the mirror, leading to the red shift of the bonding dipole plasmon mode.

Compared to the SERS peak intensity, plasmons impact the background continuum in a different way, showing NPoM geometry dependence. In the aforementioned mechanism for the origin of the background, the inelastic light scattering of electrons requires extra momentum provided by the localized plasmon. This localized plasmon can be easily achieved and greatly enhanced in an NPoM plasmonic nanocavity. Thus, the background continuum is predicted to increase with tight confinement of plasmons, which in a certain way depends on the change in geometry. Although not explicitly discussed, the SBR of SERS spectra reported in the literature shows variation due to the modification in plasmonic nanostructures, such as nanovoids [38,39], nanospheres [10,22,28], and nanocubes [40,41]. Moreover, increasing the nanoparticle size can enhance the near-field intensity in the gap region of nanocavities [23]. Specifically, for the nanocubes, the localized electric field intensity for the bonding dipole plasmon resonance mode shows a clear increase at the center of the NCoM geometry with the increase in edge length [24]. This may be responsible for the nanoparticle-geometry-dependent variation of the SERS background. On the other hand, localization of plasmons is also responsible for the in-coupling of light to metal nanostructures and the out-coupling of the radiation from metal. Since the inelastic light scattering arises from photoexcited electrons in the metal, the plasmon providing strong optical fields in the plasmonic nanocavity will enhance the light scattering with electrons. Similarly, the confinement of incoming light will enhance the Raman scattering of molecules in the gap region. For the out-coupling of the radiation from the metal, the spectra of plasmon resonance will decide the energy range of electrons contributing to the background. This may explain why a greater red shift in the plasmon resonance due to the change in nanoparticle geometry gives a more pronounced background continuum in SERS. However, the plasmon enhancement effect alone is insufficient to explain the irreversibility of the background after laser irradiation with higher power. We note that the conformation change of molecules within the metal junction can affect the conductivity of the junction [42,43]. The irreversible background upon laser exposure with high power may arise from the increased conductivity of gold due to the formation of a conductive bridge between the Au nanoparticles and the mirror [36]. Actually, in our previous paper, we found that the PL of aggregated Au nanospheres can be enhanced by high-power continuous-wave laser irradiation, due to laser-induced photothermal welding of the aggregates [44]. A detailed investigation into the influence of conductivity on light emission of Au in NPoM geometry and the SERS spectra would be interesting, but is, however, beyond the scope of this work.

## 4. Conclusions

In summary, by performing laser exposure on NPoM plasmonic nanocavities formed with different nanoparticle geometries, we investigated the changes in SERS peak intensity of the spacer molecules and the continuous background. Our findings indicate that the laser-induced conformation change of the spacer molecules reduces the gap height and enhances the plasmon coupling in the NPoM systems, resulting in an enhancement in the Raman scattering from molecules. With the increase in the nanoparticle size, the background continuum shows a more significant increase than the SERS peak intensity, resulting in a lowering SBR. We suggest that plasmon-enhanced inelastic light scattering of electrons in metal is responsible for the background in SERS, which shows a clear dependence on the nanoparticle geometry. The SERS spectra among plasmonic nanocavities with a larger nanoparticle size show a higher irreversible background continuum after laser exposure, which may highlight the role of the increased conductivity of the Au nanoparticle and the mirror due to laser exposure. Clarifying the dependence of the SERS background on the geometry of plasmonic nanocavities should suggest new routes to modify the signal-to-background ratio of SERS spectra and thus improve the SERS sensitivity.

## Data Availability

Data underlying the results are contained within the article or Supplementary Materials.

## Supplementary Materials

The following supporting information can be downloaded at: https://www.mdpi.com/article/10.3390/nano14010053/s1, Figure S1: Scanning Electron Microscope (SEM) images; Figure S2: Size distribution of Au nanocubes and nanospheres; Figure S3: Schematic of the experimental setup for surface-enhanced Raman scattering; Figure S4: Schematic of the experimental setup for dark-field scattering spectroscopy; Figure S5: Typical evolution of SERS spectra of spacer molecules; Figure S6: Laser power-dependent SBR of the SERS spectra.
