# Supplementary material for: The Geometry of Nanoparticle-on-Mirror Plasmonic Nanocavities Impacts Surface-Enhanced Raman Scattering Backgrounds
Source: Nanomaterials (Basel). 2023 Dec 24;14(1):53. doi: 10.3390/nano14010053 (PMC10780556; doi:10.3390/nano14010053)
Supplement: Supplementary file 1 [file nanomaterials-14-00053-s001.zip › nanomaterials-2784866-supplementary.pdf]

nanomaterials

Supplementary Materials

The Geometry of Nanoparticle-on-Mirror Plasmonic Nanocavities Impacts Surface-Enhanced Raman Scattering Backgrounds

Zixin Wang [1,2], Wenjin Zhou [1,2], Min Yang [1,2], Yong Yang [1,2], Jianyong Hu [1,2], Chengbing Qin [1,2], Guofeng Zhang [1,2], Shaoding Liu [3], Ruiyun Chen [1,2,*] and Liantuan Xiao [1,2,4,*]

1 State Key Laboratory of Quantum Optics and Quantum Optics Devices, Institute of Laser Spectroscopy, Shanxi University, Taiyuan 030006, China
2 Collaborative Innovation Center of Extreme Optics, Shanxi University, Taiyuan 030006, China
3 Key Laboratory of Advanced Transducers and Intelligence Control System, Ministry of Education, Taiyuan University of Technology, Taiyuan 030024, China
4 College of Physics, Taiyuan University of Technology, Taiyuan 030024, China
* Correspondence: chenry@sxu.edu.cn (R.C.); xlt@sxu.edu.cn (L.X.)

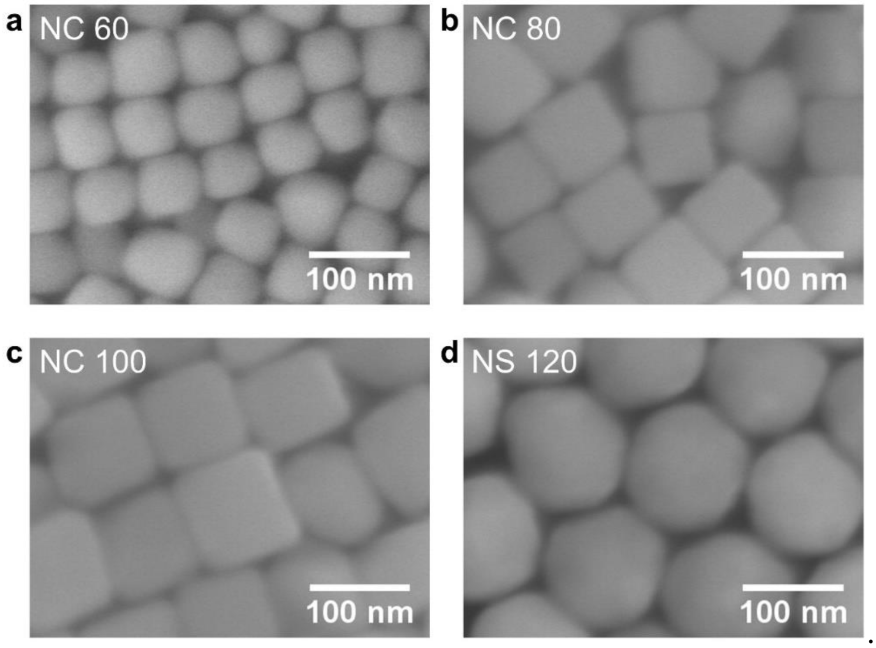

Figure S1. Scanning Electron Microscope (SEM) images of Au nanocubes and Au nanospheres for **(a)** NC 60, **(b)** NC 80, **(c)** NC 100, and **(d)** NS 120.

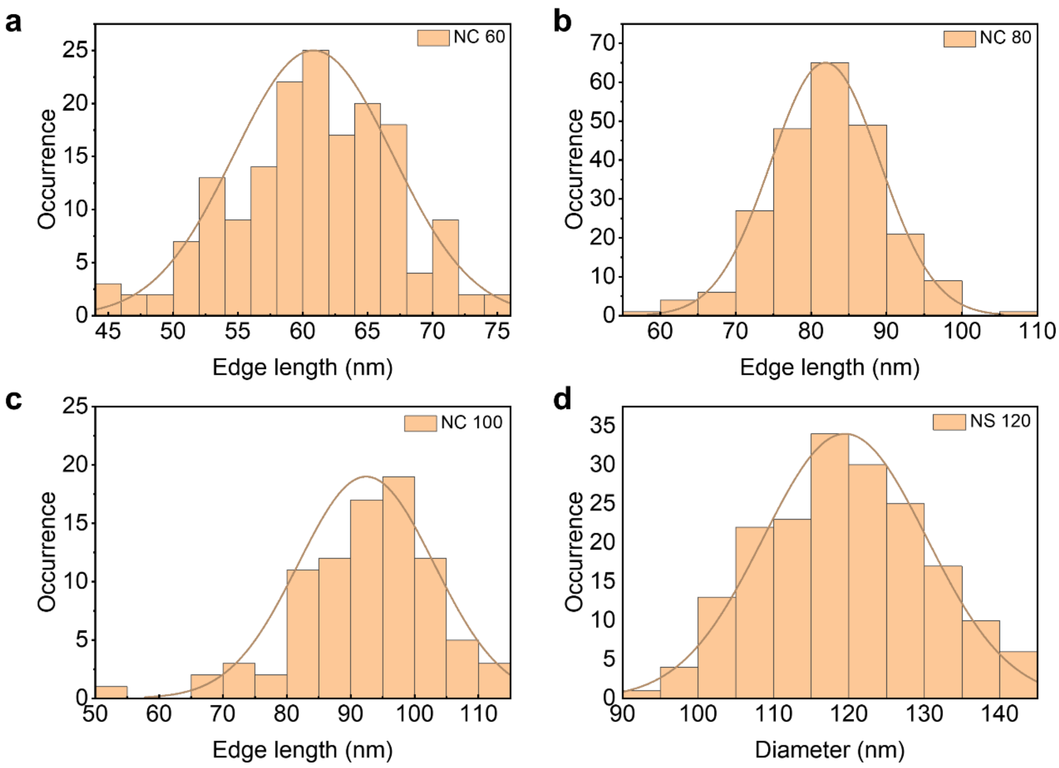

Figure S2. Size distribution of Au nanocubes and nanospheres. **(a)** NC 60 has a size distribution centered at 60.81 nm with a width of 6.13 nm; **(b)** NC 80 has a size distribution centered at 81.90 nm with a width of 7.24 nm; **(c)** NC 100 has a size distribution centered at 92.43 nm with a width of 10.64 nm; **(d)** The nanospheres has a size distribution centered at 119.49 nm with a width of 10.84 nm.

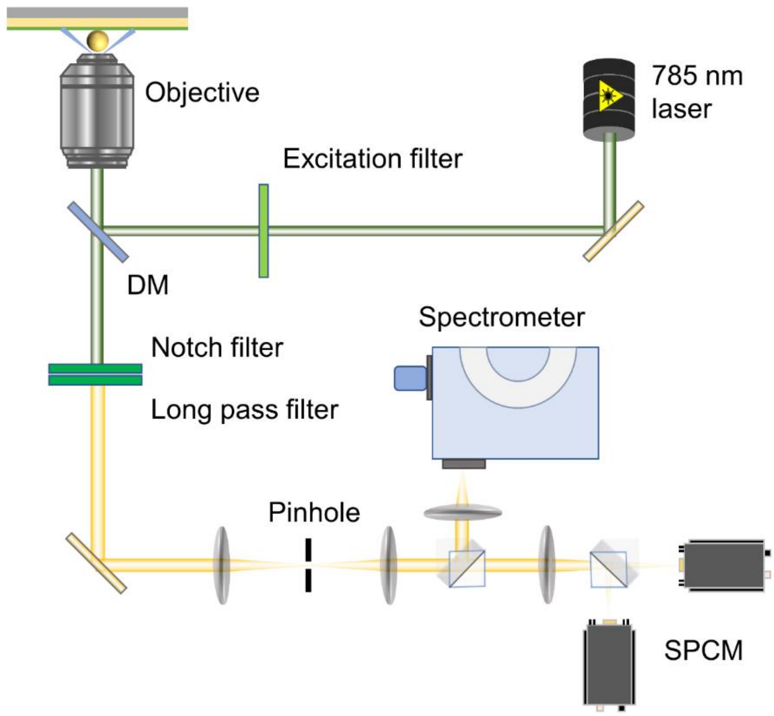

Figure S3. Schematic of the experimental setup for surface-enhanced Raman scattering.

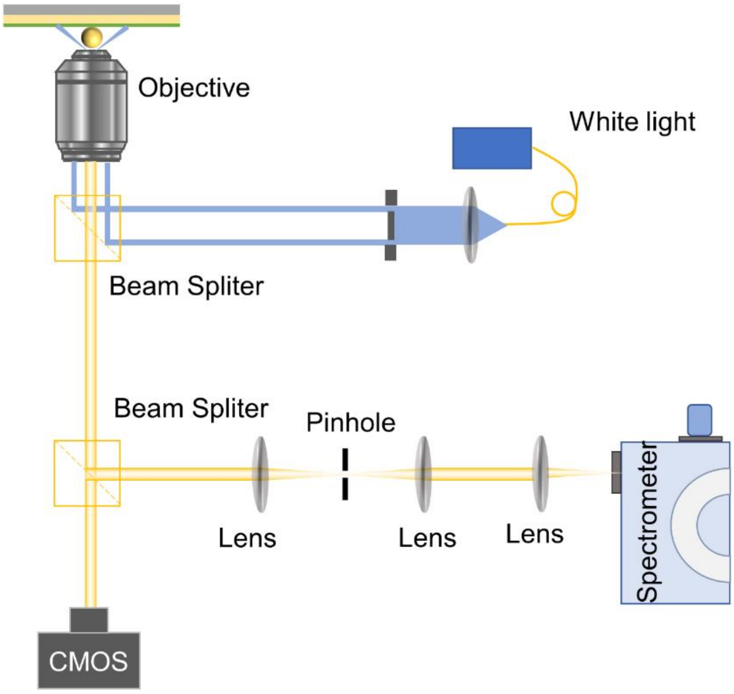

Figure S4. Schematic of the experimental setup for dark-field scattering spectroscopy.

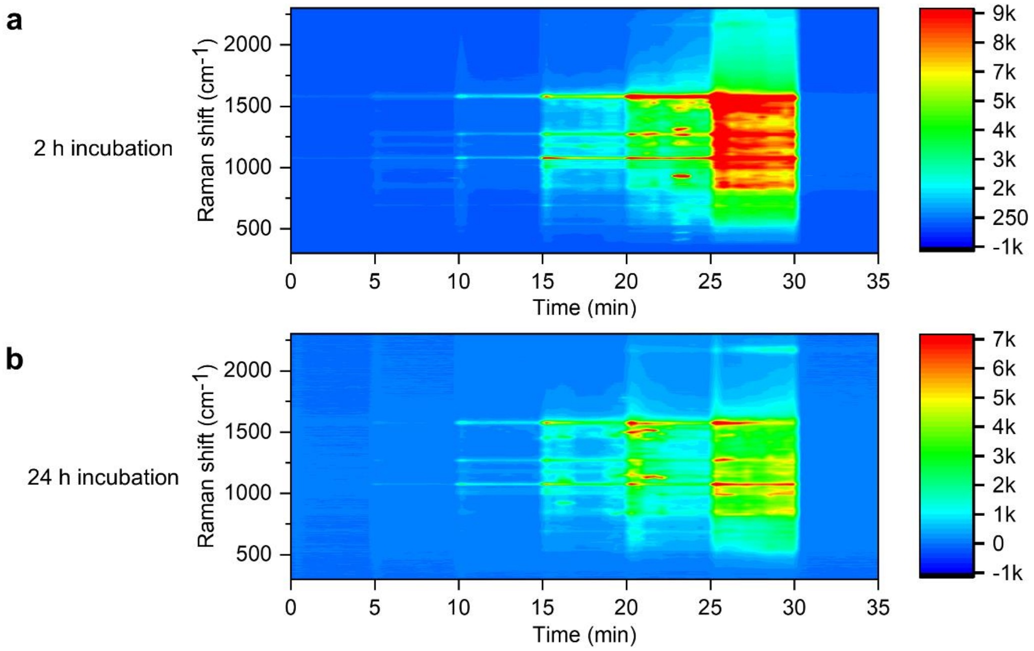

Figure S5. Typical evolution of SERS spectra of spacer molecules prepared with 2 h (a) and 24 h (b) incubation time, among NS 120 plasmonic nanocavity systems, upon laser exposure with laser power of 26.4 µW (initial), 129.4 µW, 564 µW, 1.38 mW, 2.25 mW, 4.5 mW, and 26.4 µW (final). The acquisition time for each power is 5 min.

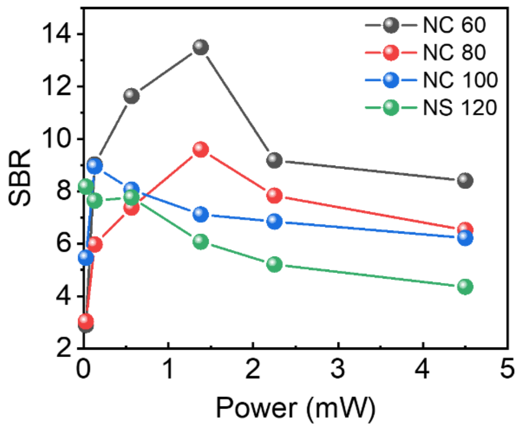

Figure S6. Laser power-dependent SBR of the SERS spectra averaged over all the investigated samples of BPhT molecules in NPoM plasmonic nanocavities with nanoparticle geometries of NC 60, NC 80, NC 100, and NS 120.
